# Evaluation of fennel seed meal in broiler chickens' diets: impacts on performance, carcass traits, digestive enzymes, intestinal microbiota, blood metabolites, and economic feasibility

**DOI:** 10.5194/aab-68-531-2025

**Published:** 2025-08-15

**Authors:** Mohammed E. Ghonime, Fathy Abdelazeem, Reham A. M. Ali, Mohamed M. Hamed, Waleed A. Hassan, Layla A. Almutairi, Mohammed A. Alqahtani, Suad H. Almasoudi, Mahmoud Alagawany

**Affiliations:** 1 Department of Animal and Poultry Production, Faculty of Agriculture and Natural Resources, Aswan University, Aswan, Egypt; 2 Department of Poultry Production, Faculty of Agriculture, Ain Shams University, Egypt; 3 Department of Animal and Poultry Production, Faculty of Agriculture, Beni Sweif University, Egypt; 4 Department of Biology, College of Science, Princess Nourah bint Abdulrahman University, P.O. Box 84428, Riyadh 11671, Saudi Arabia; 5 Department of Biology, College of Science, King Khalid University, 61413 Abha, Saudi Arabia; 6 Department of Biology, College of Sciences, Umm Al-Qura University, Makkah 21955, Saudi Arabia; 7 Poultry Department, Faculty of Agriculture, Zagazig University, Zagazig 44511, Egypt

## Abstract

The present study evaluated the effects of fennel seed meal (FSM) on performance, carcass characteristics, lengths of digestive tract parts (cm), intestinal enzyme activity, intestinal microbiology, blood parameters, and economic efficiency in broiler chickens. One hundred 44 unsexed, 1 d old broiler chicks (Ross) were divided into four groups, each with 36 chicks, at random and into four replicates, each with 9 chicks. The first group was fed the basal diet (control group), while the second, third, and fourth groups (T1–3) were fed a diet with 5 %, 10 %, and 20 % fennel seed meal, respectively. Dietary treatments significantly affected all performance parameters at 5 weeks of age. Total protein, albumin, globulin, aspartate aminotransferase (AST), total cholesterol, and triglycerides significantly affected the results, while the alanine aminotransferase (ALT) and albumin–globulin ratios did not change substantially. The results revealed no significant difference in the activity of amylase, trypsin, and lipase (U dL^−1^) between the treatments. The chymotrypsin enzyme experienced a notable impact. There was a significant increase in the total digestive tract, small intestine, and cecum length (cm) in birds fed FSM compared to the control group. Moreover, including FSM in broiler diets increased lactic acid bacteria and reduced coliforms and clostridia. Net return and economic efficiency were increased with increasing FSM levels in broiler diets. It is concluded that FSM can be used in up to 20 % of broiler chicken diets to reduce feed costs without adverse effects on broilers' growth performance and public health.

## 1 Introduction

A critical area of agriculture is poultry farming, which is distinguished by its high intensity and quick expansion. Over the past 40 to 50 years, there has been significant growth in poultry production and consumption (Daghir et al., 2021; Castro et al., 2023). This trend is predicted to continue, particularly in emerging nations, making chicken meat the most important source of meat protein for the world's expanding population. Chicken meat is so popular everywhere because it is inexpensive, has excellent nutritional and sensory qualities, is simple to prepare, and is not restricted by religion (Petracci et al., 2019).

One of the most critical challenges in poultry production feed costs is thought to be the primary expense of raising chickens, accounting for 65 %–75 % of total costs. According to Fasuyi (2005), numerous studies aim to reduce the cost of feeding to the lowest possible levels. In broiler diets, soybean meal (SBM) is frequently the main source of plant protein; however, at competitive pricing, alternative protein sources are occasionally included (Rama Rao et al., 2006).

Many authors have concluded that incorporating fennel seeds into poultry feed offers numerous benefits for the health and growth of the birds (Al-Sagan et al., 2020; Fatima et al., 2022; Waziri et al., 2023). This is because fennel seeds have been shown to enhance the health and productivity of chickens, protect them from various infectious illnesses, and explore innovative delivery methods for fennel seeds in poultry nutrition. The biological benefits of fennel in chickens include enhanced egg quality and development performance, increased proliferation of immune cells, and decreased oxidative stress (Khan et al., 2022). Additionally, it contains anethole, tannin, lima oil, fenchone, limonene, camphine, pinene, methyl chavicol, anisic acid, thymohydroquinone, and vitamin A. Fennel seed meal, which has a nutritional value appropriate for feeding poultry, is the result of manually extracting fennel oil from fennel seeds (Waziri et al., 2023; Rehman et al., 2025).

The present study hypothesized that fennel seed meal, as an alternative feed ingredient in poultry diets, could affect broiler performance and health. Therefore, the study aims to assess the effect of different levels of fennel seed meal as a feed alternative in broiler diets on productive performance, economic efficiency, carcass characteristics, blood parameters, and gut health.

## 2 Materials and methods

The current study was conducted from October to November 2023 at Aswan University's Department of Animal and Poultry Production, Faculty of Agriculture and Natural Resources. The protocols established by the Local Experimental Animal Care Committee were followed during the experimental procedures. The ethical approval code is ZU–IACUC/2/F/313/2023.

### 2.1 Experimental design and birds

One hundred 44 unsexed 1 d old broiler chicks were randomly distributed into 4 equal groups of 36 birds, subdivided into 4 replicate groups containing 9 chicks each. The first group was fed a control diet without fennel seed meal (FSM), and the second, third, and fourth groups were fed diets containing 5 %, 10 %, and 20 % FSM, respectively. The birds were fed in three phases: starter (0–14 d), grower (15–28 d), and finisher (29–35 d). Fennel seed meal was obtained after oil extraction from a rendering company (Hajj Younis Oil Mill, Qus City, Qena Governorate, Egypt) and ground into a fine powder using an electric mill. All ingredients including FSM were mixed to formulate the experimental diets, and all diets were nearly isonitrogenous and isocaloric, which covered the requirement of broiler chickens. The proximate composition of fennel seed meal was determined using the standard methods of the AOAC (2006). All samples were analyzed in triplicate for crude protein, fat, ash, moisture, crude fiber, calcium, and phosphorus (Table 1). The composition and calculated analysis of experimental diets are presented in Table 2 for the three phases.

**Table 1:** Proximate analysis of fennel seed meal.

Item	Fennel seed meal
Composition (%)^a^	
Crude protein	21.85
Crude lipid	30.28
Crude fiber	7.70
Calcium	0.40
Total phosphorus	0.55
Ash	7.80
ME, kcal kg^−1^ (calculated)^b^	4157

^a^ Determined according to AOAC (2006). ^b^ Calculated metabolizable energy according to Carpenter and Clegg (1956).

**Table 2:** Composition and calculated analysis of the starter, grower, and finisher experimental basal diets.

	Starter (0–14 d)				Grower (15–28 d)				Finisher (29–35 d)			
Ingredients %	C	T1	T2	T3	C	T1	T2	T3	C	T1	T2	T3
Yellow corn	52.96	50.63	47.96	41.00	55.96	54.26	51.48	45.00	61.77	59.20	56.73	50.88
Soybean meal (46 % CP)	33.00	33.00	33.00	33.67	32.10	31.00	31.00	31.00	28.00	28.00	28.00	26.36
Corn gluten meal (60)	7.00	5.60	4.10	1.10	5.00	4.20	2.75	0.19	4.20	2.80	1.40	0.00
Fennel seed meal	0.00	5.00	10.00	20.00	0.00	5.00	10.00	20.00	0.00	5.00	10.00	20.00
Soybean oil	2.40	1.55	0.75	0.00	2.80	1.70	1.00	0.00	2.80	2.10	1.00	0.00
Monocalcium phosphate	0.96	1.64	1.52	1.40	2.18	1.43	1.33	1.23	1.75	1.10	1.00	0.76
Limestone	2.50	1.36	1.35	1.33	0.88	1.25	1.20	1.21	0.55	0.82	0.82	1.00
HCL-lysine	0.28	0.30	0.32	0.37	0.21	0.25	0.27	0.30	0.12	0.13	0.15	0.10
DL-methionine	0.30	0.32	0.40	0.53	0.27	0.31	0.37	0.47	0.21	0.25	0.30	0.30
Salt	0.30	0.30	0.30	0.30	0.30	0.30	0.30	0.30	0.30	0.30	0.30	0.30
Premix^a^	0.30	0.30	0.30	0.30	0.30	0.30	0.30	0.30	0.30	0.30	0.30	0.30
Price EGP/(ton)	2221	2066	1953	1779	2142	1986	1878	1688	2024	1887	1759	1559
Calculated analysis^b^												
Metabolizable energy (kcal kg^−1^)	3017	3024	3024	3060	3050	3053	3059	3087	3105	3118	3100	3143
Crude protein (%)	23.07	23.08	23.03	23.13	21.57	21.53	21.50	21.56	19.54	19.54	19.55	19.51
Crude fiber (%)	2.53	2.86	3.17	3.80	2.53	2.82	3.14	3.75	2.46	2.78	3.09	3.66
Calcium (%)	0.97	0.97	0.97	0.98	0.87	0.88	0.87	0.89	0.65	0.65	0.65	0.67
Available phosphors (%)	0.49	0.48	0.48	0.49	0.44	0.44	0.44	0.45	0.37	0.36	0.36	0.40
Lysine (%)	1.40	1.44	1.48	1.58	1.29	1.33	1.26	1.29	1.09	1.12	1.08	1.11
Methionine (%)	0.67	0.67	0.75	0.86	0.61	0.64	0.66	0.72	0.52	0.55	0.57	0.59
Methionine + cysteine (%)	1.09	1.082	1.13	1.20	0.99	1.01	0.99	0.99	0.87	0.88	0.85	0.88

^a^ The premix contains 15 000 000 I.U. vitamin A and 3 000 000 I.U. vitamin D in 50 g portions for every 3 kg. Vitamin E, 3000 mg K3 vitamin, 3000 mg vitamin B1, 8 000 mg. Vitamin B2, 4000 mg. Vitamin B6, 20 mg, pantothenic acid, 15 000 mg, niacin, 60 000 mg, folic acid, 1500 mg, biotin, 200 000 mg, and vitamin B6 vitamin C 700 g of choline chloride, 80 g of manganese, 80 g of zinc, 60 g of iron, 10 g of copper oxide, 1 g of iodine, and 0.2 g of millennium; 3 kg of premix per ton of feed was the inclusion rate, with CaCO_3_ being used as a carrier up to 3 kg. ^b^ The experimental diets were calculated according to Brazilian feedstuffs (2017).

### 2.2 Bird management

Feed was provided freely in stainless-steel feeders in the form of pellets for grower and finisher feed and mash for starter feed, all based on experimental diets. All birds were reared under the same managerial conditions with free access to water and feed during the experimental period. Excreta were removed daily to ensure all birds were kept under the same managerial, hygienic, and environmental conditions throughout the experimental period. All birds were vaccinated by a drinking-water-based vaccine for Newcastle at 7 d, for Gambaro at 14 d, and for Lasota twice at 18 and 28 d. All vaccines for animal health were obtained from the Veterinary Serum and Vaccine Research Institute (VSVRI) in Egypt.

### 2.3 Measurements and procedures

#### 2.3.1 Productive performance

Live body weight (LBW) was determined by a balance with a readability of 0.01 g. Body weight gain (g) (BWG), feed consumption (g) (FC), feed conversion ratio (FCR) (g feed / g gain), and mortality rate were also determined, in addition to the performance index and European productive efficiency factor. According to North (1981), the performance index (PI) is calculated as (final LBW [kg] / FCR), while Emmert (2000) defines the European productive efficiency factor (EPEF) as (final LBW [kg] ⋅ survival rate %) / (FCR ⋅ rearing periods [days]).

#### 2.3.2 Carcass characteristics

After 35 d of breeding, four chicks from each treatment group were chosen. The chickens were kept off feed and water for 3 h before their sacrifice. After recording their live weights, the chickens were immediately sacrificed by severing the jugular vein and allowed to bleed completely following the scientific method. Their percentages of carcass, liver, heart, empty gizzard, spleen, bursa, and abdominal fat were recorded. The length of the digestive tract parts was also recorded in centimeters.

#### 2.3.3 Digestive enzyme activity (U dL^−1^)

At the end of the 35th day of age, individual intestinal contents were collected in dry, clean tubes from four chicks within each treatment. We quantitatively determined the levels of amylase, lipase, trypsin, and chymotrypsin. All the digestive enzyme activity of the samples was determined according to Nitsan et al. (1991).

#### 2.3.4 Cecum microbiology

At the end of each experiment, one bird from each group was randomly selected for digesta sampling in the cecum. The cecum's contents were used to study the microbiological flora in the Ain Shams University's Faculty of Agriculture microbiology laboratory. Total bacteria, coliforms, and lactic acid bacteria were counted. Samples were taken in plastic 20 mL tubes and cooled until incubation. The samples were processed quickly after collection. Samples were weighed (1 g) and serially diluted in 0.9 % saline, and 1 mL of each sample was dispensed and spread on selective media in Petri dishes. After shaking, 10 mL of the extract was taken for further dilutions. From diluted extracts (10^1^–10^6^), plates were prepared with a specific medium for each studied microorganism. Brilliant Green Agar media was used for *Salmonella*, and MacConkey agar plates were used for coliforms. Microbial suspension from each sample dilution was transferred through the pour plate method (Quinn et al., 2002) and incubated at 37 °C for 24 h. The pathogens were identified by growing on specific media and through biochemical tests. Accordingly, the incubation medium, MRS agar, was used for lactic acid bacteria (LAB). LAB counts of the cecum contents were obtained at 30 °C following 3 d of incubation. After that, the colonies were counted through the colony counter. The total colony count was expressed as log10 cfu g^−1^ of contents and determined by multiplying the reciprocal of the dilution factor by the average number of colonies. The microbial counts were determined as logarithmic colony-forming units (cfu) per gram of sample.

#### 2.3.5 Blood plasma parameters

Levels of total protein, albumin, cholesterol, triglycerides, and aspartate aminotransferase (AST) and alanine aminotransferase (ALT) enzyme activity were calorimetrically determined using commercial diagnostic kits (produced by Spectrum Company, Egypt).

#### 2.3.6 Economic efficiency

The economic efficiency of broiler chicks was determined. The cost of litter feed was determined using local market feed prices, and economic efficiency was calculated based on prices in 2023.

### 2.4 Statistical analysis

Data on performance, carcass and digestive enzymes, and plasma biomarkers in broiler chickens were analyzed with a generalized linear model. The SAS user's guide (2001) provided the following model as a basis for analysis: $Y^{ij}=T_{\mu }+T_{i}+e_{ij}$ , where $Y^{ij}=$ observation, $T_{\mu }=$ overall mean, $T_{i}=$ FSM effect, and $e_{ij}=$ random error. The treatment’s effect ( i=1 –4) and the random error are represented. The mean values of each experimental and control group were compared using Tukey’s multiple-range tests.

**Table 3:** Effect of dietary treatments on productive performance of broiler chicks during 0–35 d of age.

Items	Treatments				SEM	Significance
	Control	T1	T2	T3		
Live body weight (g)						
0 d	44.25	44.35	44.50	42.20	0.30	N.S.
35 d	1996.39^a^	1961.28^a,b^	1941.24^a,b^	1906.89^b^	11.81	*
Body weight gain (g d^−1^)						
0–35 d	1950.14^a^	1916.93^a,b^	1899.74^a,b^	1864.69^b^	11.75	*
Feed consumption (g/feed)						
0–35 d	2921.25^b^	3045^a^	3066.25^a^	2962.5^b^	18.08	*
FCR (g feed g^−1^ gain)						
0–35 d	1.50^b^	1.59^a^	1.61^a^	1.59^a^	0.10	*
PI	133.37^a^	123.50^b^	120.12^b^	119.94^b^	1.71	*
EPEF	381.04^a^	352.86^b^	343.20^b^	342.70^b^	4.89	*
MR	0	0	0	0		

Means in the same row with different superscripts are significantly ( p>0.05 ) different. N.S.: non-significant. ^∗^: significant. Control: basal diet, T1: feed with 5 % FSM (fennel seed meal), T2: feed with FSM 10 %, and T3: feed with FSM: 20 %.

## 3 Results

### 3.1 Growth performance

Table 3 shows the effect of FSM on growth performance. At 5 weeks of age, the results revealed significant differences between treatments. There was no significant difference between birds fed on the basal diet, T1, and T2; there was a significant difference between the T3 groups, which had the lowest LBW, and the control groups, which had the highest LBW.

There was a significant difference among treatments during the 5-week fattening period (0–35 d). Live body weight and weight gain were decreased in birds fed 20 % FSM compared to the control group and other treatments. The results showed that birds fed on the basal diet (control groups) consumed the least feed compared to other treatments (T1–3). The corresponding values for feed consumption during all experimental periods (0–35 d) ranged between 3045, 3066.25, and 2962.5 g, with significant differences compared to the control group (2921.25 g).

The results revealed significant differences in FCR values between treatments during 0–35 d of age. There was no significant difference between birds fed on FSM (T1–3); the range of values was ( 1.59:1.61 ). The best value of FCR was obtained with the control group (1.50).

The data showed a significant difference in PI and EPEF among treatments during the studied period (0–35 d). Chicks fed control diets reflected the highest figures of PI and EPEF compared to other treatments. The corresponding values for PI ranged between (133.37) and EPEF (381.04), while chicks fed on 5 % FSM diets showed figures of 123.50 and 352.86, respectively. The differences among treatments were significant. In the same order, chicks fed FSM (T2–3) showed the lowest figures of PI or EPEF compared with those fed on control diets. The corresponding values for PI and EPEF ranged for chicks fed on 10 % FSM between 120.12 and 343.20, respectively. On the other hand, chicks fed on 20 % FSM diets displayed figures of 119.94 and 342.70, respectively.

Under the conditions of the present study, all chicks appeared healthy, and the total mortality rate was 0.00 % during the entire experimental period (0–35 d). There were no apparent differences between the experimental treatments and the control one.

**Table 4:** Effect of dietary treatments on carcass characteristics (%) and lengths of digestive tract parts (cm) of broiler chicks.

Items	Treatments				SEM	Significance
	Control	T1	T2	T3		
Carcass %	70.45	72.60	72.69	73.32	0.30	N.S.
Liver %	1.62	1.67	1.61	1.79	0.5	N.S.
Gizzard %	1.27	1.23	1.23	1.37	0.3	N.S.
Heart %	0.50	0.55	0.49	0.58	0.2	N.S.
Abdominal fat %	1.41^a^	1.05^a^	0.83^a,b^	0.46^b^	0.11	*
Bursa %	0.07	0.06	0.08	0.08	0.10	N.S.
Spleen %	0.09^b^	0.11^a,b^	0.14^a^	0.10^b^	0.10	*
Lengths of digestive tract parts (cm)						
Total digestive tract	192.75^b^	193.375^b^	199.875^a,b^	219.875^a^	4.85	*
Small intestine	169.25^c^	172.5^b^	176.5^b^	198.25^a^	5.46	*
Cecum length	15.25^c^	16.875^c,b^	18.05^a,b^	19.23^a^	0.46	*

Means in the same row with different superscripts are significantly ( p>0.05 ) different. N.S.: non-significant. ^∗^: significant. Control: basal diet, T1: feed with 5 % FSM (fennel seed meal), T2: feed with FSM 10 %, and T3: feed with FSM: 20 %.

### 3.2 Carcass characteristics

The data presented in Table 4 showed no significant difference in all carcass traits among treatments, except for the spleen and abdominal fat percentage. Birds fed 20 % FSM showed significantly lower abdominal fat percentage than the other groups. Birds fed 10 % FSM achieved the best spleen organ value compared to the other groups. The results showed significant increases in total digestive tracts, small intestine, and cecum length in birds fed different dietary treatments (T1–3) compared to the control group.

**Table 5:** Effect of dietary treatments on intestinal enzymes activity (U dL^−1^) of broiler chicks.

Items	Treatments				SEM	Significance
	Control	T1	T2	T3		
Amylase	3.84	2.99	4.02	3.73	0.17	N.S.
Lipase	10.79	9.17	9.24	11.31	0.43	N.S.
Trypsin	32.88	27.78	29.90	31.16	1.56	N.S.
Chymotrypsin	42.83^a^	31.04^b^	32.93^b^	26.58^b^	2.38	

Means in the same row with different superscripts are significantly ( p>0.05 ) different. N.S.: non-significant. Control: basal diet, T1: feed with 5 % FSM (fennel seed meal), T2: feed with FSM 10 %, and T3: feed with FSM: 20 %.

### 3.3 Intestine enzyme activity (U dL^−1^)

Table 5 shows that the levels of amylase, trypsin, and lipase (U dL^−1^) did not change significantly when birds were fed a diet with FSM compared to a basal diet. The results revealed significant differences in chymotrypsin activity between chicks fed different dietary treatments from FSM compared to the control group.

**Table 6:** Effect of dietary treatments on cecal microbiology (CFU g^−1^) in broiler chicks.

Items	Treatments			
	Control	T1	T2	T3
Total counts $\times 10^{5}$	450	358	315	298
Clostridium $\times 10^{1}$	3	–	–	–
Coliforms $\times 10^{3}$	5	4	–	–
Lactic acid bacteria $\times 10^{4}$	120	150	210	202

Control: basal diet, T1: feed with 5 % FSM (fennel seed meal), T2: feed with FSM 10 %, and T3: feed with FSM: 20 %.

### 3.4 Cecum microbiology (CFU g^−1^)

The results obtained in Table 6 showed that increasing FSM levels from 0 % to 20 % in broiler diets decreased the total bacterial count from 450 to 298 CFU g^−1^ and increased the lactic acid bacteria from 120 to 202 CFU g^−1^. Using FSM in broiler diets significantly reduced the count of clostridium and coliforms compared to the control group.

**Table 7:** Effect of dietary treatments on some blood parameters.

Items	Treatments				SEM	Significance
	Control	T1	T2	T3		
Total protein (g dL^−1^)	4.68^a^	3.91^b^	3.79^b^	3.77^b^	0.13	*
Albumin (g dL^−1^)	2.07^a^	1.75^b^	1.83^a,b^	1.73^b^	0.05	*
Globulin (g dL^−1^)	2.61^a^	2.04^b^	2.09^b^	2.05^b^	0.09	*
Albumin / globulin (ratio)	0.80	0.86	0.88	0.85	0.03	N.S.
AST (U L^−1^)	59.07^a^	32.27^b^	29.45^b^	28.61^b^	4.68	*
ALT (U L^−1^)	11.40	11.75	10.32	10.23	0.58	N.S.
Total cholesterol (mg dL^−1^)	88.28^a^	67.82^b^	60.01^b^	51.76^b^	0.44	*
Triglycerides (mg dL^−1^)	46.02^a^	40.45^b^	32.33^b^	31.76^a,b^	2.18	*

Means in the same row with different superscripts are significantly ( p>0.05 ) different. N.S.: non-significant. ^∗^: significant. Control: basal diet, T1: feed with 5 % FSM (fennel seed meal), T2: feed with 10 % FSM, and T3: feed with FSM 20 %.

### 3.5 Blood metabolites

The data presented in Table 7 illustrate the effect of feeding broiler chickens diets with different levels of FSM on plasma protein profiles. Significant decreases in total protein, globulin, and albumin were recorded compared with those of the control group. These findings, in turn, have not influenced the A/G ratio, as no significant increase was observed compared with the control group. Table 7 shows no significant differences in ALT levels between the control group and the other treatments regarding liver function, as shown by plasma AST and ALT enzymes. Still, there were significant differences in AST levels between the control group and the other treatments. The current experiment showed a significant effect of FSM substitution in broiler diets up to 35 d of age on reducing the cholesterol level in plasma, suggesting that FSM supplementation might play a role in broiler lipid metabolism. Unfortunately, little information has been published on the effects of FSM supplementation on blood lipid metabolites in broiler diets. The current trial also indicated that broiler diets supplemented with FSM significantly decreased triglyceride levels in plasma.

**Table 8:** Effect of dietary treatments on economic efficiency of broiler chicks.

Items	Treatments			
	Control	T1	T2	T3
AFC (g)	2755	2790	2708	2690
Feed cost/chicken (EGP)	58.65	55.25	50.46	45.06
Total cost/chicken (EGP)	98.65	95.25	90.46	85.06
LBW (g)	1870.33	1885.66	1837.79	1803.5
Total return (EGP)	140.27	141.42	137.83	135.26
Net return (EGP)	41.62	46.17	47.37	50.2
EE %^a^	42.18	48.47	52.36	59.01
Relative EE %^b^	100	114.91	124.13	139.90

Control: basal diet, T1: feed with 5 % FSM (fennel seed meal), T2: feed with FSM 10 %, and T3: feed with FSM 20 % diet. Live body weight (LBW) is equal to average feed consumption (AFC). The local price of 1 kg of chicken LBW was EGP 75. ^a^ EE (economic efficiency) = net return/total chicken cost ×100 . ^b^ Relative EE is equal, assuming EE of the control sample is 100 %.

### 3.6 Economic efficiency

Table 8 shows the effect of dietary FSM on economic evaluation, where the use of FSM in broiler diets improved their economic feasibility. Compared to birds fed control diets from 0–35 d of age, broiler chicks fed FSM with varying growth (T1–3) exhibited economic efficiency (EE) values of 48.47, 52.36, and 59.01, respectively, outperforming the control group by 42.18 %. The groups fed on diets with FSM (T1–3) exhibited improvements in relative economic efficiency (REE) values (14.91 %, 24.13 %, and 39.90 %, respectively) compared to the control groups. This has increased economic efficiency and lowered feed costs because fennel seed meal is inexpensive.

## 4 Discussions

According to the results, broiler diets containing varying amounts of FSM as a source of protein had a negative effect on body weight, body weight gain, feed consumption, and feed conversion ratio at the lowest, 10 %, and 20 % levels of FSM. These results agreed with Safaei-Cherehh et al. (2020), who found that adding fennel extract to the diet made them eat less, which led to weight loss. On the other hand, Hosseini et al. (2015) found that layers did not change how much feed they ate when they ate fennel seed. Liu et al. (2021) also found that adding different amounts of fennel seed powder (FSP) to their diet did not significantly affect the broilers' body weight, average daily gain, daily feed intake, or feed-to-gain ratio. Also, Al-Sagan et al. (2020) found that in normal temperatures, the addition of 1.6 % and 3.2 % fennel seed powder to the diets did not affect the body weight gain or feed intake of broilers; nevertheless, under chronic heat stress, the results revealed substantial changes in the broiler's performance. On the contrary, Ragab (2007), Mohammed and Abbas (2009), and Lemrabt et al. (2018) recorded that feed consumption, feed conversion ratio, body weight, and body weight gain improved when FSM was added to broiler diets. Nassar et al. (2024) stated that dietary supplementation with fennel seed would be a useful nutritional strategy to enhance poultry production.

Carcass characteristics are an important economic indicator in the poultry industry and a valuable criterion for measuring net meat production capacity in broiler chickens, evaluating meat production yield, and assessing the impact of nutrition on poultry. Carcass and offal ratios are key indicators for measuring meat production performance in poultry. In the present study, dietary FSM did not affect carcass characteristics substantially, except that abdominal fat was significantly higher in the T3 group. The results of carcass characteristics in this study were in line with those obtained by Al-Sagan et al. (2020), who reported that the supplementation of 1.6 % and 3.2 % FSP did not show differences in carcass characteristics. Similar results were reported by Liu et al. (2021) and Ghiasvand et al. (2021). In the same context, Mohammed and Abbas (2009) and Cengiz et al. (2016) found that the broiler carcass traits were unaffected by adding 100 mg kg^−1^ of fennel oil to the diet. Ghiasvand et al. (2021) found that broiler carcass traits were unaffected by adding 200 mg kg^−1^ of fennel essential oil to the diet. On the contrary, Cengiz et al. (2016), Gharaghani et al. (2015), Ragab (2007), Henda (2014), and Al-Sagan et al. (2020) observed that feeding broiler diets with 1.2 % and 3.2 % fennel seed powder increased the size of the gizzard and the whole digestive tract. Cengiz et al. (2016) showed that adding 100 mg kg^−1^ of fennel essential oil to broiler feeds enhanced the weight, size, and overall health of the digestive tract of the animals. When fennel was added to the diets of laying hens, the carcass traits increased by 10 and 20 g kg^−1^. Gharaghani et al. (2015) found that in Japanese quails' diets, adding 0.5 %–1 % fennel seed increased carcass traits (Ragab, 2007). Adding 0.75 g kg^−1^ of fennel seed meal in Japanese quail diets increased carcass traits (Henda, 2014).

In this study, birds fed FSM diets achieved the best immune system performance, particularly in the spleen. These results may be attributed to the biological benefits of fennel in chickens, which increased immune cell proliferation and reduced oxidative stress (Khan et al., 2022). It was shown that feeding birds' diets containing fennel seed meal (FSM) positively impacted the total digestive tract length, the length of the cecum, and the small intestinal tract. These results align with those obtained by Liu et al. (2021), who found that adding fennel seed powder to the diet positively affected broiler chickens' digestion and absorption capabilities and their carcass traits and intestinal histological features. This improvement in intestinal morphology facilitated healthy and efficient development in Cobb broiler chickens. Liu et al. (2021) found that in the group treated with fennel seeds, the jejunum's weight and length were significantly greater than those of the other groups.

Data showed a significant decrease in intestinal chymotrypsin activity in FSM diets compared to the control group. These results disagree with Henda (2014), Hadavi et al. (2017), and Safaei-Cherehh et al. (2020), who found fennel seed makes you hungry, boosts your body's digestive enzymes, and sets off an immune response. Malhotra (2012) discovered that fennel seed contains active ingredients and essential oils like anethole and estragole. These ingredients help the body digest food by increasing bile acid production and digestive enzymes such as protease, lipase, amylase, and maltase. This may be why birds that ate fennel seed also ate more food. Hadavi et al. (2017) reported that fennel extract supplementation in poultry diets can significantly change enzyme activity. Studies have shown that fennel extract can lower the activities of hepatic enzymes in the serum, such as alanine aminotransferase and aspartate aminotransferase (Liu et al., 2021; Fatima et al., 2022; Barakat et al., 2023).

The current study showed that using FSM in broiler diets increases lactic acid bacteria and decreases coliforms and clostridium. This could be due to the potential of FSM levels in diets to alter the growth and composition of the microbiota. Many researchers agreed with this result (Sadeghi et al., 2015; Yadav and Jha, 2019). Wang et al. (2015) found in their study that the effectiveness of fennel seeds as growth promoters in broiler diets had improved the microorganisms in the small intestinal tract. In this study, the total cholesterol and triglycerides were significantly lower in the FSM groups than in the control group. This suggests that FSM enhances lipid profiles in broiler chickens, which could be attributed to improving public health.

It is well established that feed cost contributes the largest portion of the variable costs of poultry production. It represents about 65 %–75 % of the total costs (El-Deek et al., 2020). Therefore, introducing a cheaper feed source into the poultry diet would reduce high feed costs and improve the economic efficiency of poultry farms. In the current study, the FSM-based diets were found to be the most economical, due to the lower market price of FSM compared to the expensive feeds. In light of the economic evaluations, it would be appropriate for all future research efforts to determine the optimal level of fennel or its derivatives in poultry diets, not to ignore the profitability of poultry farming, and to conduct a cost–benefit analysis of data collected more regularly.

## 5 Conclusion

From the previous results, we conclude that using FSM as a nontraditional feedstuff can improve net revenue, economic efficiency, and plasma lipid profile while decreasing intestinal infections, resulting in an overall improvement in their health state. Moreover, up to 20 % of FSM could be used as an alternative feedstuff in broiler chicken diets.

## Data Availability

The data presented in this study are available on request from the corresponding author.
